# The joint effects of planetary *β*, topography and friction on baroclinic instability in a two-layer QG model

**DOI:** 10.1017/jfm.2025.10172

**Published:** 2025-05-26

**Authors:** Miriam F. Sterl, André Palóczy, Sjoerd Groeskamp, Michiel L. J. Baatsen, Joseph H. LaCasce, Pål E. Isachsen

**Affiliations:** 1 NIOZ Royal Netherlands Institute for Sea Research, Texel, The Netherlands; 2 Institute for Marine and Atmospheric Research, Utrecht University, Utrecht, The Netherlands; 3 National Oceanography Centre, Liverpool, United Kingdom; 4 Department of Geosciences, University of Oslo, Oslo, Norway; 5 Norwegian Meteorological Institute, Oslo, Norway

## Abstract

The quasi-geostrophic two-layer model is a widely used tool to study baroclinic instability in the ocean. One instability criterion for the inviscid two-layer model is that the potential vorticity (PV) gradient must change sign between the layers. This has a well-known implication if the model includes a linear bottom slope: for sufficiently steep retrograde slopes, instability is suppressed for a flow parallel to the isobaths. This changes in the presence of bottom friction as well as when the PV gradients in the layers are not aligned. We derive the generalised instability condition for the two-layer model with nonzero friction and arbitrary mean flow orientation. This condition involves neither the friction coefficient nor the bottom slope; even infinitesimally weak bottom friction destabilises the system regardless of the bottom slope. We then examine the instability characteristics as a function of varying slope orientation and magnitude. The system is stable across all wavenumbers only if friction is absent and if the planetary, topographic and stretching PV gradients are aligned. Strong bottom friction decreases the growth rates but also alters the dependence on bottom slope. Thus the often mentioned stabilisation by steep bottom slopes in the two-layer model only holds in very specific circumstances and thus probably plays only a limited role in the ocean.

## 1 Introduction

Mesoscale eddies are ubiquitous in the ocean (Ferrari & Wunsch 2009; Storer *et al*. 2022) and play a key role in the global ocean circulation, ecosystems and the climate system (e.g. Wolfe & Cessi 2010; Zhang & Vallis 2013; Gnanadesikan *et al*. 2015; Busecke & Abernathey 2019). Most global climate models do not have sufficient horizontal resolution to resolve mesoscale eddies; instead, the effects of eddies are parameterised (e.g. Eden & Greatbatch 2008; Hallberg 2013; Hewitt *et al*. 2017; Kjellsson & Zanna 2017; Yankovsky *et al*. 2022). Good understanding of eddy dynamics is highly relevant to accurately parameterising them. The main generation mechanism of mesoscale eddies is baroclinic instability (Gill *et al*. 1974; Robinson & McWilliams 1974). Baroclinic instability may occur when the isopycnals of the background density fields are sloped, providing a reservoir of available potential energy which can be converted to eddy energy. Baroclinic instability occurs almost everywhere in the ocean (Smith 2007*b*; Tulloch *et al*. 2011; Feng *et al*. 2021). To understand eddy dynamics, it is thus vital to understand baroclinic instability.

Theoretical quasi-geostrophic (QG) models for baroclinic instability were first developed by Charney (1947) and Eady (1949). Phillips (1951, 1954) introduced a simplified version: the two-layer QG model. In this model, two fluid layers with homogeneous properties are stacked on top of each other, with different densities and mean flows. The mean flow difference (vertical shear) causes the isopycnal interface between the two layers to tilt due to thermal wind balance. The two-layer QG model incorporates the most important features of baroclinic flows (Flierl 1978). Thus, the simplicity and controllability make the model very suitable for studying baroclinic instability, and for linear stability analysis in particular. Although the properties of the fully developed nonlinear eddy field may differ from those predicted by linear stability theory (Early *et al*. 2011; Berloff & Kamenkovich 2013a,b; Wang *et al*. 2016), understanding the linear dynamics can still shed light on the role of different physical properties in the system.

In its most basic version, the two-layer model describes a zonal mean shear on an *f*-plane over a frictionless flat bottom. However, the model can be modified to include planetary *β* and bottom topography. These both come into the two-layer QG model in the form of potential vorticity (PV) gradients, although they are not dynamically equivalent (Deng & Wang 2024). PV gradients are relevant for eddy dynamics as they suppress eddy mixing (Nakamura & Zhu 2010; Klocker *et al*. 2012; Sterl *et al*. 2024). In the context of linear analysis, both planetary and topographic PV gradients are found to suppress growth rates and stabilise long waves (Blumsack & Gierasch 1972; Pedlosky 1987; Wang *et al*. 2016; Leng & Bai 2018). Linear bottom slopes in particular have an important effect: where steep prograde slopes (shear and topographic wave propagation in the same direction; isopycnals and isobaths in opposite directions) only suppress wave growth rates, steep retrograde slopes (shear and topographic wave propagation in opposite directions; isopycnals and isobaths in the same direction) beyond a ‘critical slope’ value stabilise the flow completely, suppressing all baroclinic instability (e.g. Tang 1976; Ikeda 1983; Steinsaltz 1987; Pavec *et al*. 2005; Poulin & Flierl 2005; Chen & Kamenkovich 2013; LaCasce *et al*. 2019). This result follows from the Charney-Stern-Pedlosky criterion, which states that the PV gradient must change sign between the layers for instability to occur (Charney & Stern 1962; Pedlosky 1963, 1964).

The stabilising effect of steep linear retrograde slopes is a well-studied phenomenon. It is typically studied in the context of an inviscid zonal flow, with all the PV gradients in the system aligned. However, there are also studies that describe the effect of including bottom friction and of varying the orientations of the mean shear and the bottom slope. In this study, we combine all of these effects in a single model to study the instability properties. We review some known results below.

We first consider the relative orientation of the mean shear and the bottom slope. It is known that on the *β*-plane, even a weak nonzonal component of the mean shear can destabilise an otherwise stable system (Kamenkovich & Pedlosky 1996; Walker & Pedlosky 2002; Arbic & Flierl 2004b; Smith 2007a; Hristova *et al*. 2008). Moreover, even a small zonal slope can destabilise the system (Chen & Kamenkovich 2013; Khatri & Berloff 2018, 2019) and increase cross-stream eddy fluxes (Boland *et al*. 2012; Brown *et al*. 2019). Leng & Bai (2018) introduced a wavenumber coordinate system to study the baroclinic instability of nonzonal currents. They showed that small differences in the orientation of the mean shear and bottom slope result in different instability characteristics. However, in the case studies they describe, the bottom slope is still perpendicular to the mean shear. In other words, the topographic and stretching PV gradient are still aligned, though not with the planetary PV gradient.

Next, we consider the effect of including bottom friction. Numerical models require bottom friction to balance the energy budget and in some cases to limit the inverse energy cascade and thereby achieve realistic levels of mesoscale activity (Arbic & Flierl 2004a; Wang *et al*. 2016; Radko *et al*. 2022). However, bottom friction alters the baroclinic instability properties, in particular destabilising flows that are inviscidly stable. Although bottom friction damps the total eddy energy in the system, it can also increase the eddy available potential energy (APE) while damping the eddy kinetic energy. This in turn increases energy conversion from the background APE to the eddy APE. Thus it is possible that as a net effect, more energy is released than without bottom friction (Lee 2010a). This effect is referred to as ‘dissipative destabilisation’ or ‘frictional instability’ and has been studied using various methods (Holopainen 1961; Romea 1977; Pedlosky 1983; Weng & Barcilon 1991; Lee & Held 1991; Rivière & Klein 1997; Krechetnikov & Marsden 2007, 2009; Lee 2010a,b; Swaters 2010; Willcocks & Esler 2012).

However, the joint effect of bottom friction and bottom slopes in the two-layer QG model has received less attention. Weng (1990) discussed the Eady model with a sloping bottom and Ekman layers at both the top and the bottom and showed that for weak nonzero frictional strength, the critical slope value for instability is extended to a larger value. Swaters (2009) considered a hybrid planetary geostrophic-quasigeostrophic model and showed that flows over a sloping bottom are destabilised by a bottom Ekman boundary layer, for any finite Ekman number.

In this study, we transform the two-layer model equations to a wavenumber coordinate system, following Leng & Bai (2018) but adding bottom friction (Section 2). In Section 3, we use the transformed equations to derive instability conditions for a two-layer system with arbitrarily oriented shear and slope. For the inviscid case, we derive a generalised version of the Charney-Stern-Pedlosky criterion, demonstrating the stabilising effect of planetary *β* and steep retrograde slopes for aligned flows (Section 3.1). For nonzero friction, however, the instability condition becomes independent of the bottom slope (Section 3.2). We then study how the instability characteristics – growth rate, wavenumber and propagation direction of the most unstable mode – depend on the orientations of the stretching PV, planetary PV and topographic PV gradients relative to each other and on the frictional strength (Section 3.3). We conclude and discuss our results in Section 4.

## 2 Model

### 2.1 Two-layer model equations

We study a two-layer quasi-geostrophic (QG) model on a *β*-plane, over a linear bottom slope, with linear bottom friction and forced externally at the surface (e.g. Pedlosky 1987; Leng & Bai 2018). We index the upper layer with *i* = 1 and the lower layer with *i* = 2. The two layers have densities *ρ*_*i*_ and thicknesses *H*_*i*_; the total depth is *H* ≡ *H*_1_ + *H*_2_. The total flow field is the sum of a uniform and stationary background field and an eddy field that varies in space and time. We assume that the eddy field is of small amplitude compared to the background field. The background potential vorticity (PV) is denoted by *Q*_*i*_ and the background flow velocity vector by **U**_*i*_. Finally, the eddy PV, eddy flow velocities and eddy streamfunction are denoted by *q*_*i*_, **u**_*i*_ and *ψ*_*i*_, respectively. The QGPV equations for the two-layer system are

(2.1) $$\frac{\partial q_{i}}{\partial t}+\left(U_{i}+u_{i}\right)\cdot \nabla \left(Q_{i}+q_{i}\right)=\delta_{i1}ℱ-\delta_{i2}\mu \nabla^{2}\psi_{2},$$

where *δ*_*i j*_ is the Kronecker delta function, ℱ is the surface forcing and *μ* is the inverse frictional time scale. The eddy PV and velocities are related to the eddy streamfunctions:

(2.2a) $$q_{i}=\nabla^{2}\psi_{i}+{\left(-1\right)}^{i}F_{i}\left(\psi_{1}-\psi_{2}\right),$$

(2.2b) $$u_{i}=\left(-\partial \psi_{i}/\partial y,\partial \psi_{i}/\partial x\right).$$

Here *F*_*i*_ is the square of the inverse deformation radius in layer *i*, given by

(2.3) $$F_{i}=\frac{f_{0}^{2}}{g^{\prime }H_{i}},\;g^{\prime }=g\frac{\rho_{2}-\rho_{1}}{\rho_{2}},$$

with *f*_0_ the Coriolis parameter and *g*^′^ the reduced gravity. We write the background velocity in the upper layer as **U**_1_ = (*U*_1_, *V*_1_) and in the lower layer as **U**_2_ = (*U*_2_, *V*_2_). The vertical shears of the zonal and meridional background flow are Δ*U* = *U*_1_ − *U*_2_ and Δ*V* = *V*_1_ − *V*_2_, respectively. The (linear) bottom slope ***α*** = (*α*_*x*_, *α*_*y*_) induces a topographic PV gradient $\nabla B=\frac{f_{0}}{H_{2}}\alpha$ in the lower layer. The total background PV gradient is the sum of the stretching, planetary and topographic PV gradients:

(2.4a) $$\nabla Q_{1}=\left(-F_{1}\text{Δ}V,F_{1}\text{Δ}U+\beta \right),$$

(2.4b) $$\nabla Q_{2}=\left(F_{2}\text{Δ}V+B_{x},-F_{2}\text{Δ}U+\beta +B_{y}\right),$$

Finally, the term **u**_*i*_ ∇*q*_*i*_ in (2.1) represents nonlinear eddy-eddy interactions, which we will ignore in our linear analysis. Thus, the linearised versions of (2.1) in terms of the eddy streamfunctions are

(2.5a) $$\begin{array}{c} \left(\frac{\partial }{\partial t}+U_{1}\frac{\partial }{\partial x}+V_{1}\frac{\partial }{\partial y}\right)\left(\nabla^{2}\psi_{1}+F_{1}\left(\psi_{2}-\psi_{1}\right)\right)+\beta V_{1}\\ +F_{1}\left(U_{1}V_{2}-U_{2}V_{1}\right)+\left(F_{1}\text{Δ}U+\beta \right)\frac{\partial \psi_{1}}{\partial x}+F_{1}\text{Δ}V\frac{\partial \psi_{1}}{\partial y}=ℱ, \end{array}$$

(2.5b) $$\begin{array}{c} \left(\frac{\partial }{\partial t}+U_{2}\frac{\partial }{\partial x}+V_{2}\frac{\partial }{\partial y}\right)\left(\nabla^{2}\psi_{2}+F_{2}\left(\psi_{1}-\psi_{2}\right)\right)+B_{x}U_{2}+\left(\beta +B_{y}\right)V_{2}\\ +F_{2}\left(U_{2}V_{1}-U_{1}V_{2}\right)+\left(-F_{2}\text{Δ}U+\beta +B_{y}\right)\frac{\partial \psi_{2}}{\partial x}-\left(F_{2}\text{Δ}V+B_{x}\right)\frac{\partial \psi_{2}}{\partial y}=-\mu \nabla^{2}\psi_{2}. \end{array}$$

The lowest-order balance in (2.5a) is the generalised Sverdrup balance (Sverdrup 1947), by which surface forcing (ℱ) permits the mean flow to cross the mean PV gradient. There is no forcing in (2.5b), implying the mean flow must be parallel to the PV contours. This requires that:

(2.6) $$\frac{U_{2}}{V_{2}}=\frac{F_{2}U_{1}-\beta -B_{y}}{F_{2}V_{1}+B_{x}}.$$

Thus *U*_2_ and *V*_2_ are not independent. We will retain them for now, for generality, but will focus subsequently on the case *U*_2_ = *V*_2_ = 0.

At next order, (2.5a) and (2.5b) become homogeneous equations for the eddy streamfunctions *ψ*_*i*_ (Kamenkovich & Pedlosky 1996; Leng & Bai 2018). These are the equations we will focus on:

(2.7a) $$\begin{array}{c} (\frac{\partial }{\partial t}+U_{1}\frac{\partial }{\partial x}+V_{1}\frac{\partial }{\partial y})\left(\nabla^{2}\psi_{1}+F_{1}\left(\psi_{2}-\psi_{1}\right)\right)\\ +\left(F_{1}\text{Δ}U+\beta \right)\frac{\partial \psi_{1}}{\partial x}+F_{1}\text{Δ}V\frac{\partial \psi_{1}}{\partial y}=0, \end{array}$$

(2.7b) $$\begin{array}{c} \left(\frac{\partial }{\partial t}+U_{2}\frac{\partial }{\partial x}+V_{2}\frac{\partial }{\partial y}\right)\left(\nabla^{2}\psi_{2}+F_{2}\left(\psi_{1}-\psi_{2}\right)\right)\\ +\left(-F_{2}\text{Δ}U+\beta +B_{y}\right)\frac{\partial \psi_{2}}{\partial x}-\left(F_{2}\text{Δ}V+B_{x}\right)\frac{\partial \psi_{2}}{\partial y}=-\mu \nabla^{2}\psi_{2}. \end{array}$$

### 2.2 Dispersion relation for wave solutions

We look for plane wave solutions of the eddy streamfunctions in a doubly-periodic domain:

(2.8) $$\psi_{1,2}={\hat{\psi }}_{1,2}e^{i(kx+ly-\sigma t)},$$

where ${\hat{\psi }}_{1,2}$ denotes the wave amplitude, **K** = (*k, l*) = *k* (cos *θ*, sin *θ*) is the wavenumber vector, and *σ* is the angular frequency. The wave phase speed magnitude *c* is given by *c* = *σ*/*k*. Substituting (2.8) into (2.7) yields a matrix equation for the wave amplitudes:

(2.9) $$\left(\begin{array}{c} M_{11}\;\;\;\;\;\;\;\;\;M_{12}\\ M_{21}\;\;\;\;\;\;\;\;M_{22} \end{array}\right)(\begin{array}{c} {\hat{\psi }}_{1}\\ {\hat{\psi }}_{2} \end{array})=0,$$

with

(2.10a) $$M_{11}=c\kappa \left(\kappa^{2}+F_{1}\right)-\kappa^{2}\left(kU_{1}+lV_{1}\right)-F_{1}\left(kU_{2}+lV_{2}\right)+k\beta ,$$

(2.10b) $$M_{12}=\left(kU_{1}+lV_{1}-c\kappa \right)F_{1},$$

(2.10c) $$M_{21}=\left(kU_{2}+lV_{2}-c\kappa \right)F_{2},$$

(2.10d) $$M_{22}=c\kappa \left(\kappa^{2}+F_{2}\right)-\kappa^{2}\left(kU_{2}+lV_{2}\right)-F_{2}\left(kU_{1}+lV_{1}\right)+k\beta +kB_{y}-lB_{x}+i\mu \kappa^{2}.$$

To simplify the analysis, we introduce a wavenumber coordinate system, following the approach of Leng & Bai (2018). In this coordinate system, the unit vectors **e**_∥_ and **e**_⊥_ are parallel and perpendicular to **K**, respectively. We can then define the following projections:

(2.11a) $${\tilde{U}}_{i}=\frac{K\cdot U_{i}}{\kappa }=\frac{kU_{i}+lV_{i}}{\kappa }=U_{i}\cos\theta +V_{i}\sin\theta ,$$

(2.11b) $$\hat{\beta }=\frac{K\times \nabla f}{\kappa }=\frac{k\beta }{\kappa }=\beta \cos\theta ,$$

(2.11c) $$\hat{S}=\frac{K\times \nabla B}{\kappa }=\frac{kB_{y}-lB_{x}}{\kappa }=\frac{f_{0}}{H_{2}}\left(\alpha_{y}\cos\theta -\alpha_{x}\sin\theta \right)=\frac{f_{0}}{H_{2}}\hat{\alpha },$$

where *Ũ*_*i*_ is the projection of **U**_*i*_ on **e**_∥_ and $\hat{\beta },\;\hat{S}$ and $\hat{\alpha }$ are the projections of ∇ *f*, ∇*B* and ***α*** on **e**_⊥_, respectively. Thus the projections of the PV gradients on **e**_⊥_ are

(2.12a) $$\hat{\nabla Q_{1}}=F_{1}\text{Δ}\tilde{U}+\hat{\beta },$$

(2.12b) $$\hat{\nabla Q_{2}}=-F_{2}\text{Δ}\tilde{U}+\hat{\beta }+\hat{S},$$

where Δ*Ũ* = *Ũ*_1_ − *Ũ*_2_. The projections (2.11) simplify the matrix equation (2.9) to

(2.13) $$\left(\begin{array}{cc} c\left(\kappa^{2}+F_{1}\right)-{\tilde{U}}_{1}\kappa^{2}-F_{1}{\tilde{U}}_{2}+\hat{\beta } & \left({\tilde{U}}_{1}-c\right)F_{1}\\ \left({\tilde{U}}_{2}-c\right)F_{2} & c\left(\kappa^{2}+F_{2}\right)-{\tilde{U}}_{2}\kappa^{2}-F_{2}{\tilde{U}}_{1}+\hat{\beta }+\hat{S}+i\mu \kappa \end{array}\right)(\begin{array}{c} {\hat{\psi }}_{1}\\ {\hat{\psi }}_{2} \end{array})=0.$$

For nontrivial solutions, the determinant of the matrix in (2.14a) must be zero. This gives a quadratic equation for *c, A*_1_*c*^2^ + *A*_2_*c* + *A*_3_ = 0, which has the following solution:

(2.14a) $$c=\frac{-A_{2}\pm \sqrt{A_{2}^{2}-4A_{1}A_{3}}}{2A_{1}}\equiv \frac{-A_{2}\pm \sqrt{D}}{2A_{1}},$$

(2.14b) $$A_{1}=\kappa^{2}\left(\kappa^{2}+F_{1}+F_{2}\right),$$

(2.14c) $$A_{2}=-\kappa^{4}\left({\tilde{U}}_{1}+{\tilde{U}}_{2}\right)-2\kappa^{2}\left(F_{2}{\tilde{U}}_{1}+F_{1}{\tilde{U}}_{2}\right)+\hat{\beta }\left(2\kappa^{2}+F_{1}+F_{2}\right)+\left(\kappa^{2}+F_{1}\right)(\hat{S}+i\mu \kappa ),$$

(2.14d) $$\begin{array}{c} A_{3}=\kappa^{4}{\tilde{U}}_{1}{\tilde{U}}_{2}+\kappa^{2}\left(F_{2}{\tilde{U}}_{1}^{2}+F_{1}{\tilde{U}}_{2}^{2}\right)-\hat{\beta }\left(\left(\kappa^{2}+F_{1}\right){\tilde{U}}_{2}+\left(\kappa^{2}+F_{2}\right){\tilde{U}}_{1}-\hat{\beta }-\hat{S}-i\mu \kappa \right)\\ -(\hat{S}+i\mu \kappa )\left(\kappa^{2}{\tilde{U}}_{1}+F_{1}{\tilde{U}}_{2}\right). \end{array}$$

The solution for *c* can have both a real and an imaginary part. The real part, *c*_*r*_, denotes the eddy phase speed. The imaginary part, *c*_*i*_, multiplied by the wavenumber magnitude denotes the unstable growth rate, *σ*_*i*_ = *kc*_*i*_. The requirement for baroclinic instability is that *σ*_*i*_ > 0.

## 3 Linear stability analysis

### 3.1 Instability conditions in inviscid case

We can use the dispersion relation (2.14) to study the instability of the two-layer model. We start by considering the inviscid case (*μ* = 0). In this case, a necessary and sufficient condition for instability is that $D=A_{2}^{2}-4A_{1}A_{3}$ in (2.14a) is negative (as *A*_1_, *A*_2_, *A*_3_ are all real, $\sqrt{D}$ is the only possible source of an imaginary part of *c*). Here *D* is a polynomial in *k*, and from *D* < 0 it follows that there exist both a longwave and a shortwave cutoff for instability. To get insight into the role of the bottom slope, we derive another necessary condition for instability. We multiply the first row of (2.13) by $d_{1}{\hat{\psi }}_{1}^{*}/\left(c-{\tilde{U}}_{1}\right)$ and the second row by $d_{2}{\hat{\psi }}_{2}^{*}/\left(c-{\tilde{U}}_{2}\right)$, where *d*_*i*_ *H*_*i*_/*H* and * denotes the complex conjugate. Next we sum the two rows and find

(3.1) $$\kappa^{2}\left(d_{1}{\hat{\psi }}_{1}^{2}+d_{2}{\hat{\psi }}_{2}^{2}\right)+F{\left({\hat{\psi }}_{1}-{\hat{\psi }}_{2}\right)}^{2}+\frac{d_{1}{\hat{\psi }}_{1}^{2}}{c-{\tilde{U}}_{1}}\left(F_{1}\text{Δ}\tilde{U}+\hat{\beta }\right)+\frac{d_{2}{\hat{\psi }}_{2}^{2}}{c-{\tilde{U}}_{2}}\left(-F_{2}\text{Δ}\tilde{U}+\hat{\beta }+\hat{S}\right)=0,$$

where $F\equiv f_{0}^{2}/\left(g^{\prime }H\right)$. Both the real and imaginary part of (3.1) must vanish separately. The imaginary part of (3.1) is

(3.2) $$-c_{i}\left\{\frac{d_{1}{\hat{\psi }}_{1}^{2}}{{\left|c-{\tilde{U}}_{1}\right|}^{2}}\left(F_{1}\text{Δ}\tilde{U}+\hat{\beta }\right)+\frac{d_{2}{\hat{\psi }}_{2}^{2}}{{\left|c-{\tilde{U}}_{2}\right|}^{2}}\left(-F_{2}\text{Δ}\tilde{U}+\hat{\beta }+\hat{S}\right)\right\}=0.$$

For *c*_*i*_ ≠ 0, which is necessary for instability, (3.2) implies that

(3.3) $$\left(F_{1}\text{Δ}\tilde{U}+\hat{\beta }\right)\left(-F_{2}\text{Δ}\tilde{U}+\hat{\beta }+\hat{S}\right)<0.$$

Note that (3.3) is equivalent to $\hat{\nabla Q_{1}}\hat{\nabla Q_{2}}<0$. In other words, the component of the PV gradient perpendicular to the wavenumber vector must change sign between the layers for the flow to be unstable. This is the generalisation of the Charney-Stern-Pedlosky criterion (Charney & Stern 1962; Pedlosky 1963, 1964). A number of interesting aspects emerge from the instability condition (3.3). First, the relevant parameter for stability is the vertical velocity shear $\text{Δ}\tilde{U}$ rather than the individual layer velocities. Also, planetary PV has a stabilising effect since sufficiently large $\hat{\beta }$ will make both $\hat{\nabla Q_{1}}$ and $\hat{\nabla Q_{2}}$ positive. Note that for meridional wavenumber vectors (*θ* = *π*/2 or 3*π*/2), planetary PV cannot stabilise the flow, as $\hat{\beta }=0$ then. Finally, it follows from (3.3) that there exists a critical slope for instability:

(3.4) $${\hat{\alpha }}_{c}=\frac{f_{0}\text{Δ}\tilde{U}}{g^{\prime }}-\frac{H_{2}\hat{\beta }}{f_{0}},$$

with the necessary instability condition as follows:

(3.5) $$\begin{array}{c} \hat{\alpha }<{\hat{\alpha }}_{c}\;\;\;\;\;\;\text{if}\;\;\;\;\;\;\;\;\;\hat{\nabla Q_{1}}>0,\\ \hat{\alpha }>{\hat{\alpha }}_{c}\;\;\;\;\;\;\;\;\text{if}\;\;\;\;\;\;\;\;\;\hat{\nabla Q_{1}}<0. \end{array}$$

Thus if the bottom slope component perpendicular to **K** exceeds the critical slope, there is no instability for wavenumber vectors with wave angle *θ*. (Here ‘exceeds’ can mean either to be smaller or greater, depending on the sign of $\hat{\nabla Q_{1}}$) The term $f_{0}\text{Δ}\tilde{U}/g^{\prime }$ on the right-hand side of (3.4) represents the slope of the density interface between the two layers, projected on **e**_∥_. This means that on the *f* -plane, the instability condition (3.5) is that the bottom slope component perpendicular to **K** is less steep than the isopycnal slope component parallel to **K**. Again, this is a generalisation of a well-known result for zonal flows (e.g. Blumsack & Gierasch 1972; Mechoso 1980; Pavec *et al*. 2005; Isachsen 2011; Pennel & Kamenkovich 2014). Note that (3.5) is not a sufficient condition for instability; as mentioned above, for slopes below the critical slope, there is still only instability for a limited range of wavenumber magnitudes.

### 3.2 Instability condition with bottom friction

If *μ* ≠ 0, the situation changes, as both *A*_2_ and *A*_3_ in (2.14) have an imaginary part. As seen in the dispersion relation (2.14), including bottom friction is equivalent to adding an imaginary part to the slope parameter, $\hat{S}$. The imaginary part of *c* is now given by

(3.6) $$c_{i}=\frac{\text{Im}\left(-A_{2}\right)\pm \text{Im}(\sqrt{D})}{2A_{1}}.$$

The necessary and sufficient condition for instability is that (3.6) is positive. The imaginary part of −*A*_2_ is equal to − (*k*^2^ + *F*_1_) *μk*. To get an expression for the imaginary part of $\sqrt{D}$, we first write *D* in polar coordinates:

(3.7) $$D=re^{i\gamma }\Rightarrow \sqrt{D}=\sqrt{r}e^{i(\gamma /2+n\pi )},\;n=0,1.$$

The *n* = 0 solution of (3.7) corresponds to a positive Im $\left(\sqrt{D}\right)$, while the *n* = 1 solution yields a negative value. Thus instability implies the sum in (3.6) with *n* = 0 and the difference with *n* = 1. In either case, the condition for instability reduces to:

(3.8) $$\sqrt{r}\sin(\gamma /2)>\left(\kappa^{2}+F_{1}\right)\mu \kappa .$$

We use the half-angle formula to rewrite the instability condition as (see also Swaters 2009, 2010):

(3.9) $$2r\sin^{2}(\gamma /2)=r(1-\cos\gamma )>2{\left(\kappa^{2}+F_{1}\right)}^{2}\mu^{2}\kappa^{2},$$

or, equivalently,

(3.10) Re(D)2+Im(D)2>Re(D)+2(κ2+F1)2μ2κ2.

Squaring both sides yields

(3.11) Im(D)2>4(κ2+F1)2μ2κ2Re(D)+4(κ2+F1)4μ4κ4.

We obtain Re(*D*) and Im(*D*) from $D=A_{2}^{2}-4A_{1}A_{3}$ following (2.14). Some tedious but straightforward algebra yields the following expressions:

(3.12a) Re(D)=κ4(U˜1−U˜2)2(κ4−4F1F2)+(κ2+F1)2(S^2−μ2κ2)+2β^S^(κ2(F1−F2)+F1(F1+F2))+2κ2S^(U˜1−U˜2)(κ4+F1κ2−2F1F2)+2κ4β^(U˜1−U˜2)(F1−F2)+β^2(F1+F2)2,

(3.12b) $$\begin{array}{c} \text{Im}(D)=2\hat{S}\mu \kappa {\left(\kappa^{2}+F_{1}\right)}^{2}+2\mu \kappa^{3}\left({\tilde{U}}_{1}-{\tilde{U}}_{2}\right)\left(\kappa^{4}+F_{1}\kappa^{2}-2F_{1}F_{2}\right)\\ +2\hat{\beta }\mu \kappa \left(\kappa^{2}\left(F_{1}-F_{2}\right)+F_{1}\left(F_{1}+F_{2}\right)\right). \end{array}$$

Again, only the vertical velocity shear Δ*Ũ* = *Ũ*_1_ − *Ũ*_2_ enters the expression. Substituting (3.12) in (3.11) leads eventually to many terms cancelling out. Most notably, all terms containing the bottom slope term *Ŝ* disappear. Thus the slope can no longer stabilise the flow in the presence of a bottom Ekman layer. Finally, (3.11) reduces to:

(3.13) $$16\kappa^{4}F_{1}F_{2}\mu^{2}\left(\kappa^{2}+F_{1}+F_{2}\right)\left(\text{Δ}\tilde{U}\kappa^{2}-\hat{\beta }\right)\left(F_{1}\text{Δ}\tilde{U}+\hat{\beta }\right)>0.$$

The first portion is always positive, so for the *μ* ≠ 0 case, there is instability if and only if

(3.14) $$\left(\text{Δ}\tilde{U}\kappa^{2}-\hat{\beta }\right)\left(F_{1}\text{Δ}\tilde{U}+\hat{\beta }\right)>0.$$

Thus there can only be instability if Δ*Ũ* is nonzero. A further constraint that is necessary and sufficient for instability follows from (3.14), depending on the sign of $\text{Δ}\tilde{U}\cdot \hat{\beta }$:

(3.15a) $$\;\text{for}\;\text{Δ}\tilde{U}\cdot \hat{\beta }⩾0:\;|\text{Δ}\tilde{U}|>\frac{\left|\hat{\beta }\right|}{\kappa^{2}}$$

(3.15b) $$\;\text{for}\;\text{Δ}\tilde{U}\cdot \hat{\beta }⩽0:\;|\text{Δ}\tilde{U}|>\frac{\left|\hat{\beta }\right|}{F_{1}}$$

So there is a critical shear for instability; as long as Δ*Ũ* is greater than this critical shear, the system is unstable for all wave orientations *θ*. If Δ*Ũ* and $\hat{\beta }$ have equal signs, the critical shear is determined by the planetary PV gradient and the wavenumber, and long waves are stable. If Δ*Ũ* and $\hat{\beta }$ have opposite signs, the critical shear depends on the planetary PV gradient and the upper layer deformation radius, and there is no constraint for the wavenumber magnitude. The instability is thus no longer confined to a wavenumber range with both a longwave and shortwave cutoff, as in the inviscid case; friction destabilises the system (e.g. Holopainen 1961). Planetary PV can stabilise the flow; on an *f* -plane, or for meridional wave vectors $\hat{(\beta }\;=\;0)$, there is instability for all nonzero Δ*Ũ* at wave angle *θ*. Notably, neither the friction coefficient, *μ*, nor the bottom slope, $\hat{\alpha }$, appear in the instability condition (3.14). Frictional destabilisation happens even for infinitesimally small frictional strength, consistent with Swaters (2009, 2010). Moreover, as long as (3.14) holds, the flow is unstable for all slopes, irrespective of magnitude or orientation. Interestingly, (3.14) also holds for unstable flows in the inviscid case (this follows from combining (3.3) with *A*_3_ > 0, which must be true for *D* < 0). However, (3.14) is not sufficient for instability in an inviscid system; constraints for the wavenumber magnitude and slope must be met. By contrast, for *μ* ≠ 0, (3.14) is necessary *and* sufficient for instability. The inviscid case is thus a singular limit of the two-layer model.

**Figure 1:**
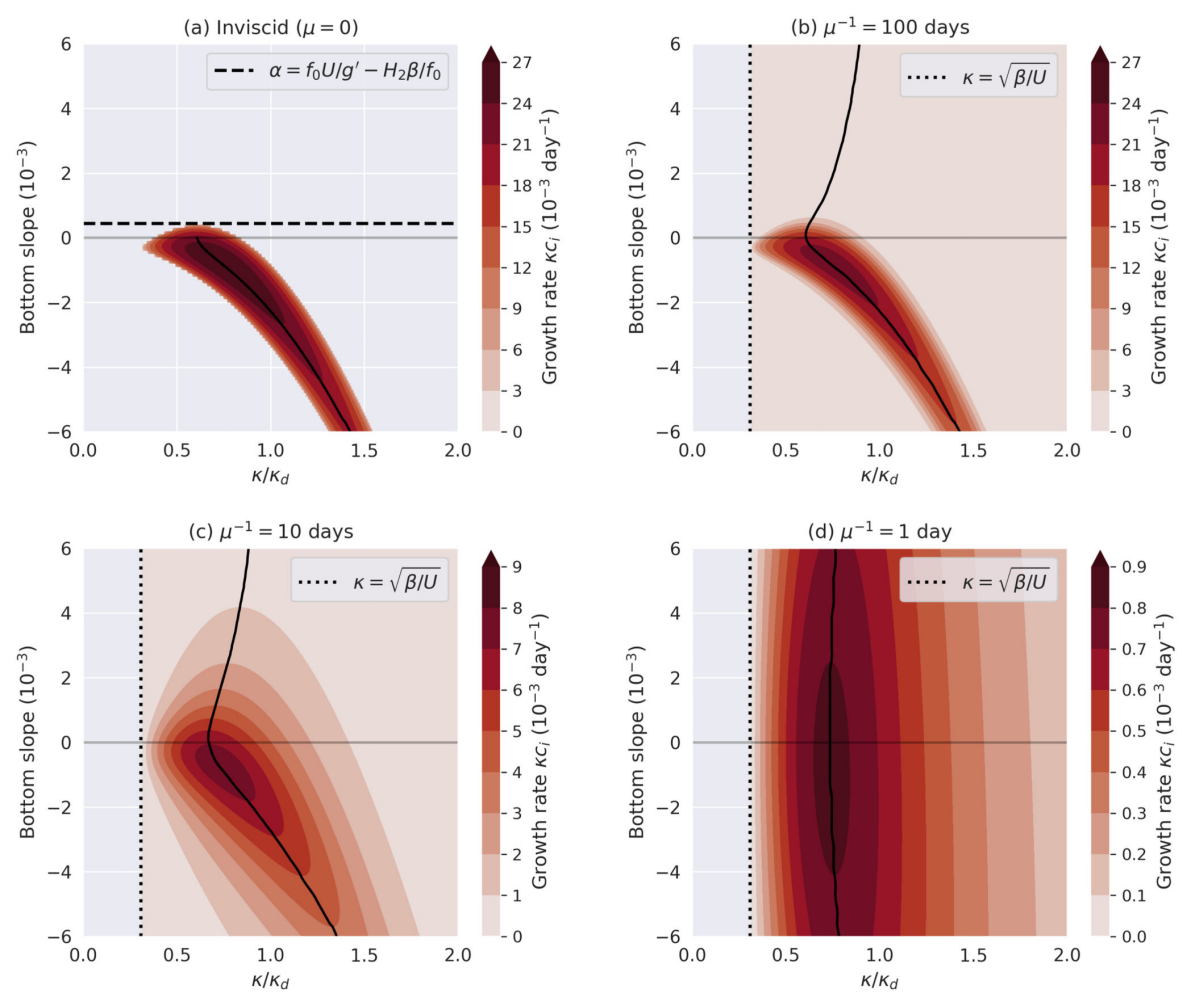
Growth rate of unstable waves as a function of wavenumber (normalised by the deformation wavenumber) for different frictional timescales, with parameters as in (3.16). Note the different colorbar ranges. The black curve in each plot shows the wavenumber that maximises the growth rate as a function of the bottom slope.

The impact of bottom slope and bottom friction on baroclinic instability is visualised in Figure 1, which shows the growth rates of unstable waves as a function of wavenumber. For this figure we consider a zonal mean shear and a meridional bottom slope, so that the planetary, topographic and stretching PV are all aligned, even without the transformation to wavenumber coordinates. We consider a zonal wavenumber vector (*θ* = 0) as this always yields the maximum growth rate. The following dimensional parameters are used, which are representative values for the ocean (e.g. Dohan & Maximenko 2010; Koltermann *et al*. 2011; LaCasce & Groeskamp 2020):

(3.16) $$\begin{array}{c} f_{0}=10^{-4}{\text{s}}^{-1},\beta =10^{-11}{\text{m}}^{-1}{\text{s}}^{-1},\text{Δ}U=0.04{\text{ms}}^{-1},\\ H_{1}=1000\text{m},H_{2}=4000\text{m},\rho_{1}=1027.5kg{\text{m}}^{-3},\rho_{2}=1028kg{\text{m}}^{-3}. \end{array}$$

In this configuration, the deformation radius $L_{d}=1/\kappa_{d}=1/\sqrt{F_{1}+F_{2}}$ is 20 km, and the upper layer deformation radius $L_{d1}=1/\sqrt{F_{1}}$ is 22 km. As estimates of the inverse frictional timescale *μ*^−1^ in the ocean range from the order of 1 to 100 days^−1^ (Arbic & Flierl 2004a), we test different orders of magnitude of *μ*. Furthermore, we test bottom slope magnitudes in the order of 10^−4^− 10^−3^, which capture most of the open ocean (LaCasce 2017). As we consider an eastward mean shear in the Northern Hemisphere, positive slopes (rising towards the north) are retrograde and negative slopes are prograde in this configuration. Figure 1a demonstrates the existence of a critical slope for instability in the inviscid case, resulting in a strong asymmetry between retrograde and prograde slopes (Blumsack & Gierasch 1972; Mechoso 1980). Figure 1b shows that even weak bottom friction destabilises the system for all bottom slopes and removes the shortwave cutoffs (as Δ*U* · *β* > 0 here, there is still a longwave cutoff at $\kappa =\sqrt{\beta /\text{Δ}U}$ equation 3.15), but also that the growth rates become weaker. There is still strong asymmetry between retrograde and prograde slopes: for retrograde slopes, growth rates are much weaker, and the maximum growth occurs at smaller wavenumbers than for prograde slopes. With increasing frictional strength (Figures 1c–d), the growth rates further decrease, and the asymmetry between retrograde and prograde slopes gets weaker. Thus, there is a shift from a slope-dominated to a friction-dominated regime. This can be understood from (2.14) by noting that the terms representing the topographic PV gradient and the bottom friction always appear together as the sum *Ŝ* + *iμk*; as *μ* increases, so does its relative importance over the topographic term.

**Figure 2:**
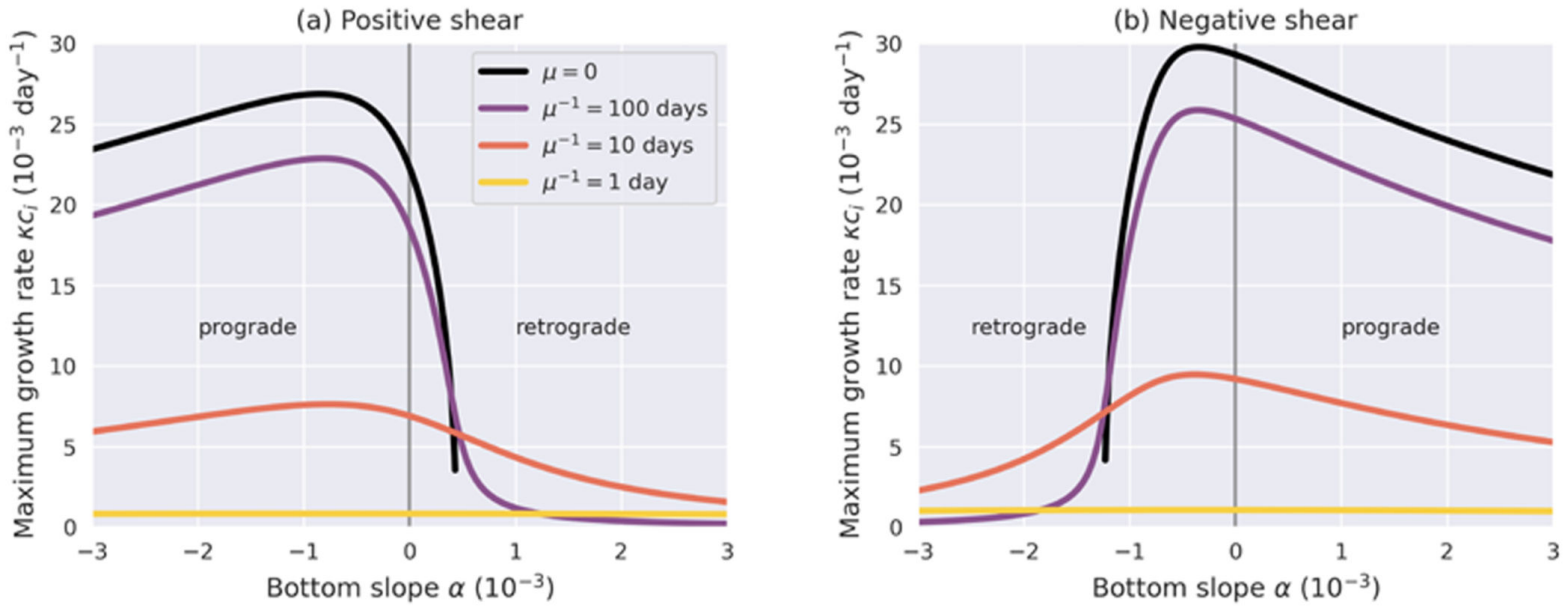
The maximum growth rate as a function of the bottom slope for different frictional strengths, for positive zonal shear Δ*U* = 0.04 m/s and negative zonal shear Δ*U* = −0.04 m/s, and the other model parameters as in (3.16).

Figure 2 shows the maximum growth rate as a function of bottom slope, for both a positive and negative zonal shear configuration. For no or weak friction, the most unstable growth rate depends strongly and asymmetrically on the bottom slope. The growth rates are much higher for prograde slopes than for retrograde slopes in this parameter configuration. For stronger, but realistic friction, the growth rate curves flatten, illustrating the shift from the slope-dominated to the friction-dominated regime. The growth rates are very low for strong friction. Hence, even though the system is unstable in an analytical sense, the unstable modes only grow very slowly for strong friction.

### 3.3 Instability characteristics for varying orientations of the PV gradients

**Figure 3:**
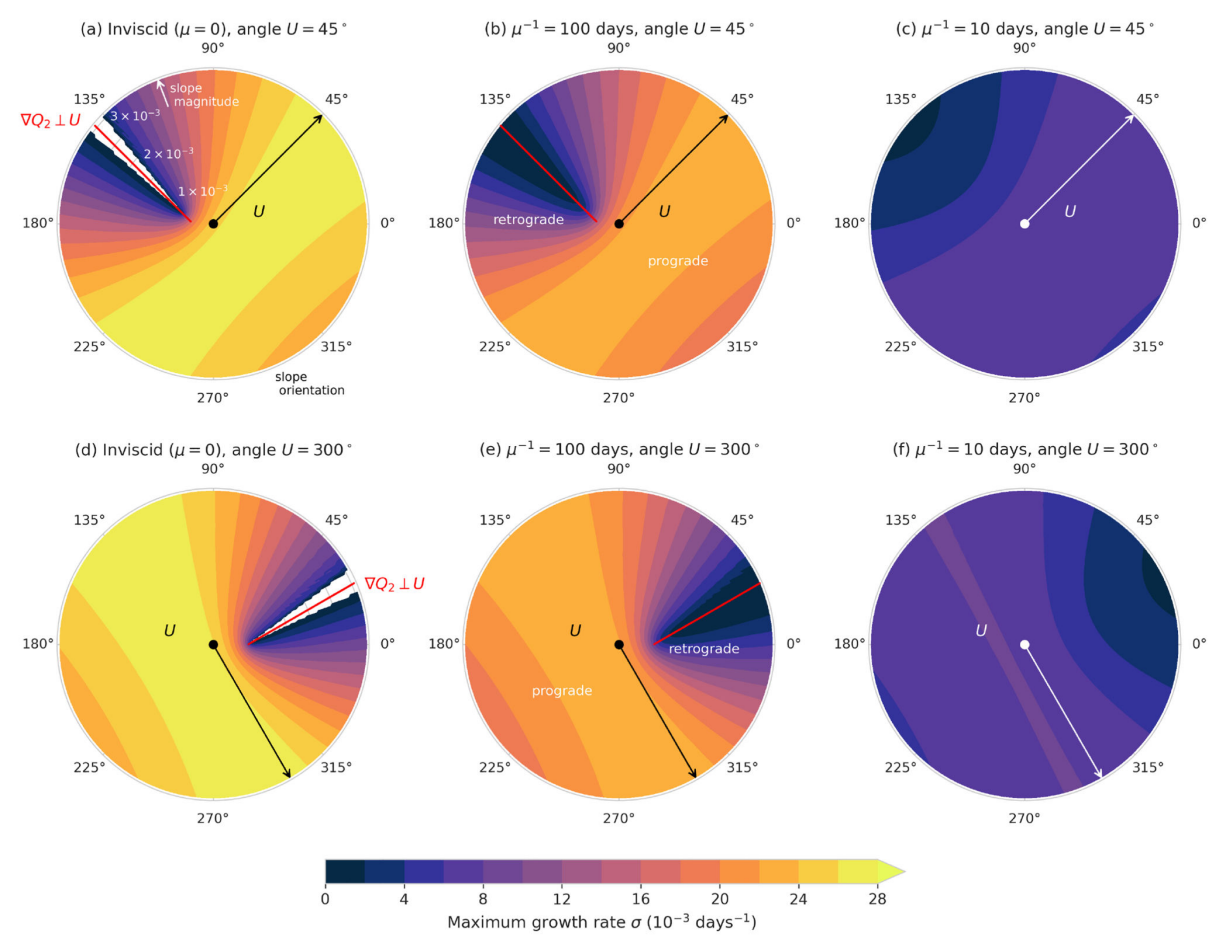
The most unstable growth rate as a function of the bottom slope vector for different orientations of the mean shear and different frictional strengths. The axes indicate the magnitude and orientation of the slope. The direction of the shear vector is indicated by the arrow in each panel and the shear magnitude is 0.04 m/s; the other model parameters are as in (3.16). The black dot marks the origin of the plot. The red lines in panels (a), (b), (d) and (e) indicate the orientation of the slope at which the lower layer PV gradient is perpendicular to the shear, as a function of the slope magnitude.

We now consider how the instability characteristics of the two-layer model change for varying orientations of the mean shear and the topography. For simplicity, we set **U**_2_ = 0 so that the shear vector is equal to **U**_1_ ≡ **U**. Figure 3 shows the most unstable growth rate as a function of the bottom slope vector, for shear vectors making an angle of 45°(top row) or 300°(bottom row) with the zonal direction. In these plots, the distance to the origin is the magnitude (steepness) of the bottom slope, and the angle around the *x*-axis is the direction of the slope (i.e., the direction in which the water column becomes shallower). For each slope vector, growth rates are computed for a range of wavenumber vectors (varying magnitudes *k* and orientations *θ*), and the maximum growth rate is plotted. Shear orientations other than 45°or 300°show similar results (not shown here). Figures 3a and 3d show that in the inviscid case, the system is only stable (white region where gridlines are visible) for a very narrow range of slope vectors; namely, close to the slope for which the lower layer PV gradient ∇*Q*_2_ (equation 2.4b) is perpendicular to **U**. Note that ∇*Q*_2_ ⊥ **U** is equivalent to the planetary, topographic and stretching PV gradients all being aligned with each other. The red line in the figures indicates the slope magnitudes and orientations for which ∇*Q*_2_ ⊥ **U**. This line and the stability region do not start at the origin: only for sufficiently steep slopes can the system become stable. For both the 45°and the 300° shear angle, stability occurs for retrograde slopes, i.e. seafloors deepening towards the right of the mean shear (in the Northern Hemisphere); prograde slopes, on the other hand, are always unstable. Moreover, as soon as ∇*Q*_2_ is not perpendicular to **U** anymore, the system becomes unstable. This does not necessarily mean that the system is unstable for all wavenumber vectors – for example, it will be stable for wavenumber magnitudes outside the longwave/shortwave cutoffs, and for wavenumber orientations for which (3.3) does not hold (see for example Figure 4 in Leng & Bai (2018)). However, Figures 3a and 3d show that for ∇*Q*_2_ not perpendicular to **U**, there is always at least some wavenumber vector for which *σ*_*i*_ is positive. As seen before, Figures 3b,c,e,f demonstrate that the presence of bottom friction destabilises the system for all bottom slopes, but also makes the growth rates (much) weaker. Figures 3a-f all show that the growth rates are symmetric around the line ∇*Q*_2_ ⊥ **U**. Growth rates increase as the slope becomes more aligned with the mean shear (so the topographic PV gradient becomes more perpendicular to the stretching PV gradient), and are generally higher for prograde slopes. As friction increases, the dependency of the growth rate on the slope weakens, and with that the asymmetry between prograde and retrograde slopes: the growth rates become very weak for all slope orientations and magnitudes.

**Figure 4:**
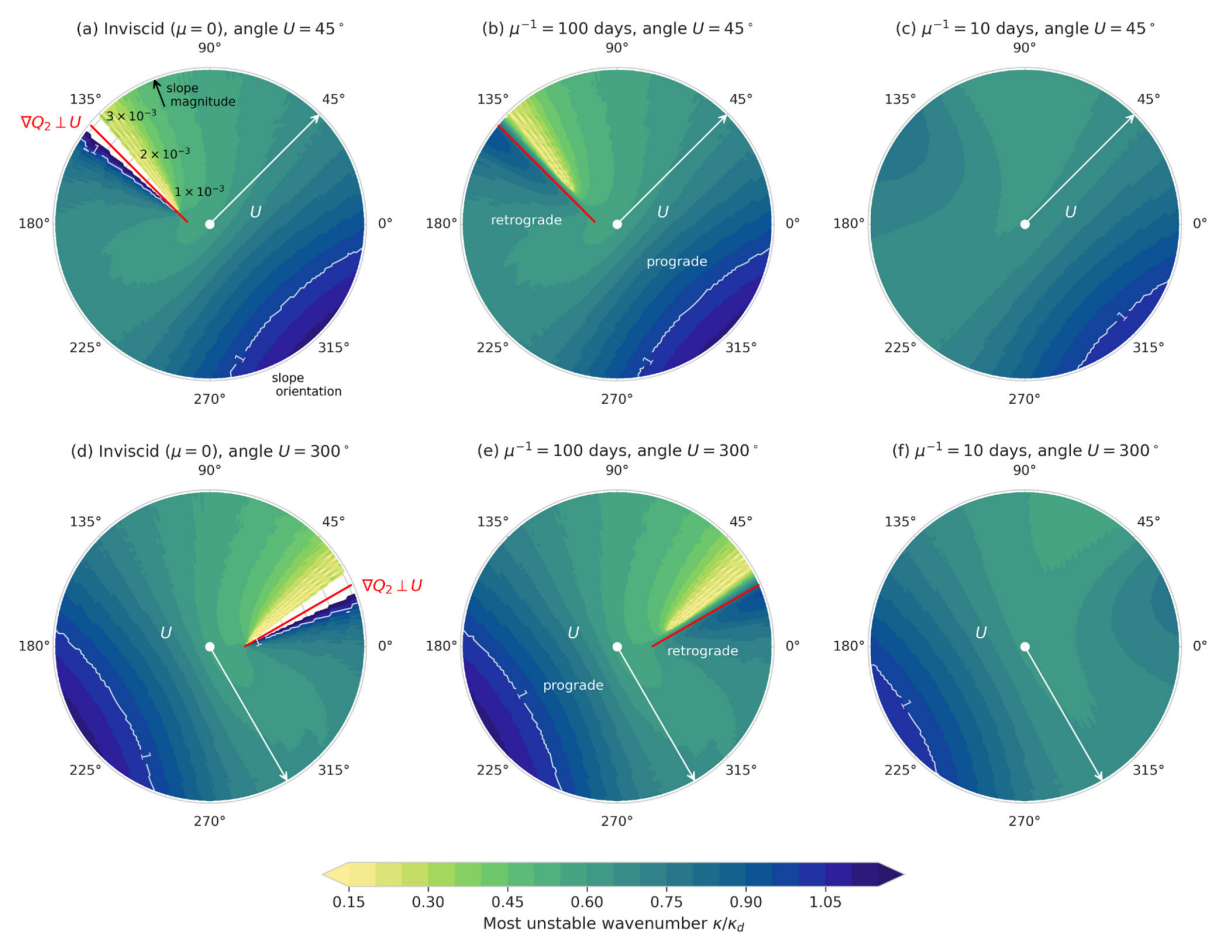
As Figure 3 but showing the most unstable wavenumber. The *k* = *k*_*d*_ contour is indicated in white.

Figure 4 shows the most unstable wavenumber as a function of bottom slope orientation and magnitude. Generally, retrograde slopes favour lower wavenumbers (larger scales), whereas prograde slopes favour higher wavenumbers (smaller scales), as in Leng & Bai (2018). An interesting feature occurs around the line ∇ *Q*_2_ ⊥ **U** in the inviscid and weak friction cases: there is a discontinuity across this line with a switch from a low wavenumber mode to a high wavenumber mode. The discontinuity goes in opposite directions for the two shear orientations considered here; the shift from low to high wavenumber occurs in the counterclockwise direction for a shear angle of 45° and in the clockwise direction for a shear angle of 300°. Other shear orientations between 0° and 270°show the same behaviour as 45°, and other shear orientations between 270° and 360° show the same as 300° (not shown here). The discontinuity across ∇*Q*_2_ ⊥ **U** is smoothed for strong friction, and the most unstable wavenumber becomes more uniform for all bottom slope magnitudes and orientations. However, the most unstable wavenumber remains asymmetric in the line ∇*Q*_2_ ⊥ **U** for retrograde slopes, as opposed to the maximum growth rate. Note that periodic patterns appear, most notably in the low wavenumber mode close to the line ∇*Q*_2_ ⊥ **U**. These are in part due to the resolution of the values of *k* and *θ* that were tested, but some periodic signal still remains even for higher resolution (not shown here). This is likely due to interactions of higher harmonics. As the patterns appear within a regime where the growth rates are very weak, they are not of great importance for the qualitative behaviour of the instability as a function of slope.

**Figure 5:**
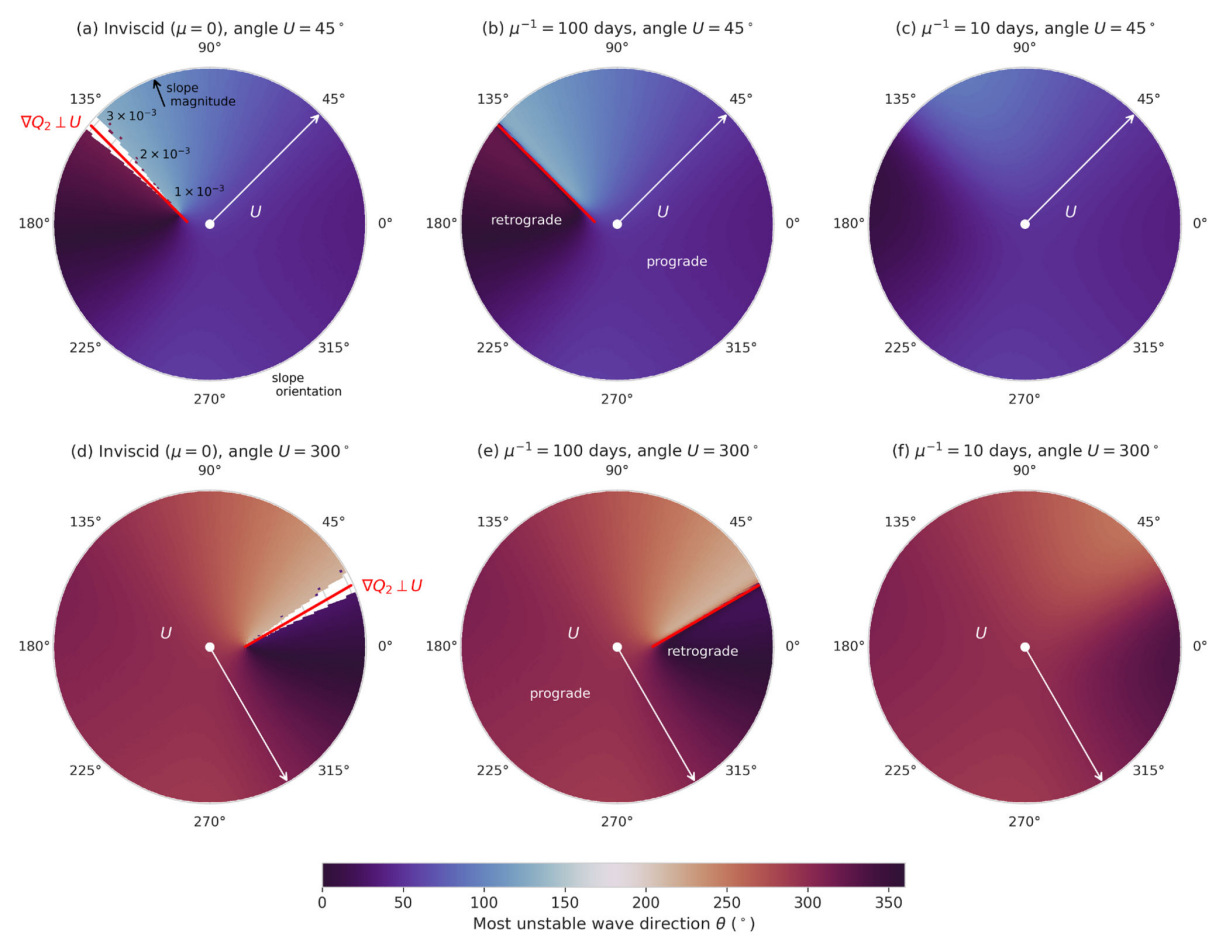
As Figure 3 but showing the propagation direction of the most unstable wave.

Finally, Figure 5 shows the propagation direction of the most unstable wave as a function of the bottom slope. If the phase speed (real part of *c*) of the most unstable wave is positive, then the propagation direction is given by the orientation *θ* of the most unstable wavenumber vector; if the phase speed is negative, then the propagation direction is exactly opposite to *θ* (shift of 180°). For the inviscid and weak friction cases, again there is a clear asymmetry between prograde and retrograde slopes. For prograde slopes, the propagation direction is close to the direction of the mean shear. For retrograde slopes, on the other hand, the most unstable wave crosses the mean flow, until the propagation direction is close to the normal direction of the mean shear around the line ∇*Q*_2_ ⊥**U**. This is in agreement with the case studies considered by Leng & Bai (2018). As in Figure 4, a discontinuity occurs across ∇*Q*_2_ ⊥**U**: the propagation direction switches by 180° across this line. This switch ensures that the most unstable wave always propagates at an acute angle to the mean shear. Over a retrograde slope with ∇*Q*_2_ · **U** > 0, the most unstable wave travels upslope, with the mean shear to its right; if ∇*Q*_2_ · **U** < 0, it travels downslope with **U** to its left. For strong friction, the discontinuity across ∇*Q*_2_ ⊥**U** disappears, as does the prograde-retrograde asymmetry: the waves all travel parallel to the mean shear.

## 4 Conclusions and discussion

We have used the two-layer QG model to study the joint effect of a planetary PV gradient, topographic PV gradients of varying orientation and magnitude, and bottom friction on baroclinic instability. A well-known instability condition of the inviscid two-layer model with a zonal mean shear and a linear meridional bottom slope is that the PV gradient must change sign between the two layers, and as a result there is a critical retrograde slope beyond which all instability is suppressed. We generalised this condition to account for other orientations of the mean shear and the bottom slope. A flow can only be stable for all wavenumbers for very specific slopes. Moreover, the instability condition no longer holds if bottom friction is present.

We first derived the instability condition with bottom friction (3.14). The system is unstable for wavenumber vectors with wave angle *θ* as long as the component of the shear parallel to the wavenumber vector is sufficiently strong; the critical shear for instability is independent of both the friction coefficient and the bottom slope. Bottom friction destabilises the system for all slope orientations and magnitudes, in line with previous studies (e.g. Holopainen 1961; Swaters 2009; Lee 2010a; Willcocks & Esler 2012). The instability condition (3.14) shows that even systems with all-positive or all-negative PV gradients can be unstable. With weak friction, the maximum growth rate is still much weaker for retrograde than for prograde slopes; as friction increases, this asymmetry disappears, and all growth rates become weaker. With strong friction, the growth rates are very weak. Hence, even though friction destabilises otherwise fully stable modes, it also suppresses the growth of the unstable modes.

We examined how the growth rates vary with the orientation of the mean shear **U** and the bottom slope. This is similar to Leng & Bai (2018), but they only considered configurations in which the bottom slope is perpendicular to the mean shear. We considered two different orientations of **U** and studied all possible orientations of the bottom slope. The system can only be stable if there is no bottom friction and the lower layer PV gradient is perpendicular to the mean shear. The slope for which this criterion holds is retrograde; prograde slopes are always unstable, and have higher growth rates. This generalises earlier findings that a system which is stable with a zonal shear and zonal isobaths can be destabilised by even a weakly nonzonal shear or weakly zonal slope (e.g. Kamenkovich & Pedlosky 1996; Chen & Kamenkovich 2013). Friction destabilises the system for all slope orientations and magnitudes, but also suppresses the growth rates. The dependency of the growth rate on the slope disappears with increasing friction. Furthermore, we found that with no or weak friction, the most unstable wave propagates along the mean flow for prograde slopes but crosses the mean flow for retrograde slopes. This agrees with the findings from Leng & Bai (2018). An interesting new finding is that there is a discontinuity in the wavenumber and propagation direction of the most unstable wave around slope vectors for which ∇*Q*_2_ ⊥**U**. Very small changes in the slope orientation can result in large changes in the instability scale and a 180° shift in the propagation direction of the most unstable mode, from parallel to antiparallel to the slope. For strong friction, the most unstable wave always travels along the mean flow.

A limitation of the two-layer QG model is that it does not account for interior PV gradients. The two layers are dynamically linked, and hence the bottom friction and bottom slope affect both layers. In contrast, Lobo *et al*. (2025) showed that while prograde flows in a three-layer QG model are reasonably represented by the two-layer model, retrograde flows are not. The reason is that retrograde flows can support surface-intensified instabilities that are almost insensitive to bottom topography, causing the flow to be more unstable than with two layers. The addition of interior PV gradients associated with an extra layer or continuous stratification might thus affect the instability conditions. An interesting case for further research would be the effect of bottom friction and misalignment of PV gradients on baroclinic instability in the three-layer QG model.

Also of interest is the impact of the type of friction on stability. The present study only considers bottom friction, but not lateral eddy friction. Moreover, we only considered linear (Ekman) friction, but many models use quadratic bottom friction instead (e.g. Chang & Held 2021; Chen 2023; Deng & Wang 2024). Linear and quadratic bottom friction have different impacts on the turbulence properties of the two-layer model (Grianik *et al*. 2004; Gallet & Ferrari 2020, 2021) and may thus affect stability in distinct ways, possibly in terms of frictional instability. As linear stability analysis is no longer possible with quadratic friction, such analysis would necessarily be numerical.

Though highly idealised, the present findings could help improve understanding of baroclinic instability in the ocean. They shed light on the dependency of instability characteristics on the bottom slope and bottom friction, and demonstrate that with increasing friction the system transitions from a slope-dominated to a friction-dominated one. This could be important for understanding the nonlinear dynamics as well (Berloff & Kamenkovich 2013a,b). For example, it would be interesting to know how the energy levels of mesoscale eddies depend on the bottom slope and bottom friction, and if they show a transition from slope-dominated to friction-dominated. Unfortunately, little is known on the strength and spatial variability of the bottom friction in the ocean, and estimates can differ by up to two orders of magnitude (Arbic & Flierl 2004a). More data on the frictional strength distribution in the global ocean is needed to properly understand which mechanism is dominant in setting the instability characteristics in the ocean. Stability caused by steep retrograde bottom slopes only holds in a very specific case: in the absence of bottom friction, when the planetary, topographic and stretching PV gradients are all aligned. Since such a specific alignment of the PV gradients is extremely rare and the seafloor is not frictionless, stabilisation by steep slopes probably does not occur in the ocean. Likewise, the ‘classical’ two-layer model with its zonal mean shear, meridional bottom slope and no friction might not be so relevant in practice when studying baroclinic instability in the ocean.

## Data Availability

The code used to create the figures in this study is available at https://github.com/MiriamSterl/TwoLayerLSA.
